# Unexplained Progressive Neurological Deficits after Corpus Callosotomy May Be Caused by Autoimmune Encephalitis: A Case of Suspected Postoperative Anti-NMDAR Encephalitis

**DOI:** 10.3390/brainsci13010135

**Published:** 2023-01-12

**Authors:** Keisuke Hatano, Ayataka Fujimoto, Keishiro Sato, Takamichi Yamamoto, Hiroshi Sakuma, Hideo Enoki

**Affiliations:** 1 Comprehensive Epilepsy Center, Seirei Hamamatsu General Hospital, 2-12-12 Sumiyoshi, Nakaku, Hamamatsu 430-8558, Japan; 2 Department of Neurosurgery, Seirei Hamamatsu General Hospital, 2-12-12 Sumiyoshi, Nakaku, Hamamatsu 430-8558, Japan; 3 Department of Brain Development and Neural Regeneration, Tokyo Metropolitan Institute of Medical Science, 2-1-6 Kamikitazawa, Setagaya-ku 156-8506, Japan

**Keywords:** anti-N-methyl-D-aspartate receptor (anti-NMDAR) encephalitis, epilepsy surgery, autoimmune encephalitis, craniotomy, case report

## Abstract

The main causes of anti-N-methyl-D-aspartate receptor (NMDAR) encephalitis are ovarian teratoma and herpes simplex virus (HSV) encephalitis. We present a rare case of suspected anti-NMDAR encephalitis caused by corpus callosotomy (CC). An 18-year-old woman with Lennox-Gastaut syndrome underwent CC. Although left hemiplegic due to cerebral hemorrhage and impaired consciousness due to cerebral venous sinus thrombosis (CVST) appeared postoperatively, anticoagulant therapy quickly improved CVST and impaired consciousness. However, various unexplained symptoms such as insomnia, hallucination, impulsivity, impaired consciousness, and a new type of drug-resistant cluster seizures gradually developed over a 2-month period. Magnetic resonance imaging revealed the gradual extension of a hyperintense area from the right frontal lobe on fluid-attenuated inversion recovery images. Intravenous methylprednisolone pulse was initiated from postoperative day (POD) 74, followed by intravenous immunoglobulin (IVIg) therapy, although white blood cell counts were normal in all three cerebrospinal fluid (CSF) examinations. After IVIg therapy, the above unexplained symptoms promptly improved. On POD 103, antibodies against NMDAR were revealed in both the serum and CSF collected before these immunotherapies. The patient was transferred to a rehabilitation hospital due to residual left hemiplegia. Psychiatric symptoms and a new onset of drug-resistant seizures may be suggestive of postoperative anti-NMDAR encephalitis, even if CSF findings are mild.

## 1. Introduction

Approximately 20–30% of patients with epilepsy reportedly cannot achieve a seizure-free state despite optimal medical treatment [1,2,3]. Some patients with drug-resistant epilepsy are candidates for epilepsy surgery [4]. Corpus callosotomy (CC) is one of the epilepsy surgeries and has also been revealed to have palliative effects, especially for drop attacks, epileptic spasms, and tonic seizures [5,6,7]. Although various complications of CC are shown, such as split-brain syndrome, hydrocephalus, temporary postoperative disturbance of consciousness, and chemical meningitis [6,8,9,10], anti-N-methyl-D-aspartate receptor (NMDAR) encephalitis after CC has not previously been reported.

Anti-NMDAR encephalitis is a type of autoimmune encephalitis caused by antibodies against the GluN1 subunit of the NMDAR [11]. Patients with anti-NMDAR encephalitis typically develop acute or subacute psychiatric and behavioral symptoms, following cold-like symptoms such as headache or fever. In addition, various symptoms, including seizures, movement disorders, impaired consciousness, and central hypoventilation, can occur, and some patients require management in an intensive care unit [12]. Despite such severe symptoms, cerebrospinal fluid (CSF) and magnetic resonance imaging (MRI) findings for anti-NMDAR encephalitis are often mild or even normal. For diagnosis, we suspect this encephalitis based on the criteria for possible autoimmune encephalitis proposed by Graus et al. [13] and need to investigate immunoglobulin (Ig)G antibodies against the GluN1 subunit in the CSF. However, relatively few facilities can investigate neuron surface antibodies, including anti-NMDAR antibodies, and some cases of autoimmune encephalitis may be overlooked.

Although several cases of non-bacterial encephalitis after craniotomy have been reported, most were attributable to herpes simplex virus (HSV) encephalitis [14,15,16,17,18]. Only one case of anti-NMDAR encephalitis has been reported as autoimmune encephalitis after epilepsy surgery, but that did not involve CC [19].

This is the first report of suspected anti-NMDAR encephalitis after CC. The diagnosis was challenging because of a pre-existing intellectual disability, mild CSF findings, and the difficulty of obtaining results for the anti-NMDAR antibody. We suggest the importance of checking the criteria for possible autoimmune encephalitis when a case shows unexplained progressive neurological deterioration after neurosurgery. Cases that meet these criteria should be investigated for neuron surface antibodies, even if CSF and MRI findings are mild.

## 2. Case Presentation

**Figure 1:**
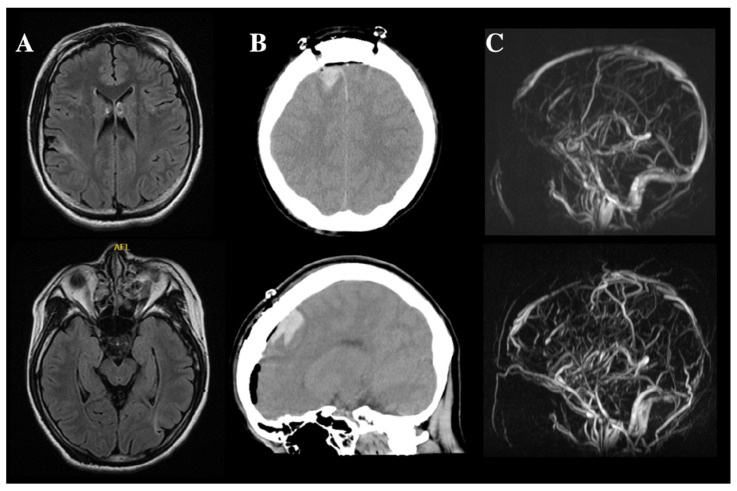
MRI and CT of the patient with anti-NMDAR encephalitis after CC. (**A**) MRI before CC revealed cortical scars at the right postcentral gyrus and left occipital lobe. (**B**) CT the day after surgery showed cerebral hemorrhage around the right supplemental motor area. At this time, mild edema was also apparent at the left frontal lobe. (**C**) Superior and inferior images represent pre- and postoperative MRA, respectively. The superior sagittal sinus and straight sinus were not evident on postoperative MRA.

An 18-year-old woman with drug-resistant epilepsy was admitted for CC due to daily seizures that persisted even after vagus nerve stimulation. There was no family history of epilepsy. The seizure types were tonic seizures, spasms, and drop attacks. She had an intellectual disability (intelligence quotient 27, Tanaka-Binet intelligence scale) and was diagnosed with Lennox-Gastaut syndrome (LGS). Preoperative MRI revealed cortical scarring in the right parietal and left occipital lobes (Figure 1A), although she had no history of stroke, contusion, or encephalitis. We performed anterior CC by the right hemispheric approach without any problems. Computed tomography (CT) immediately after the operation did not show any abnormalities. However, cerebral hemorrhage and cerebral venous sinus thrombosis (CVST) were observed soon after CC, and diverse neurological deficits progressed sub-acutely after CVST was resolved.

### 2.1. Cerebral Hemorrhage and CVST

**Figure 2:**
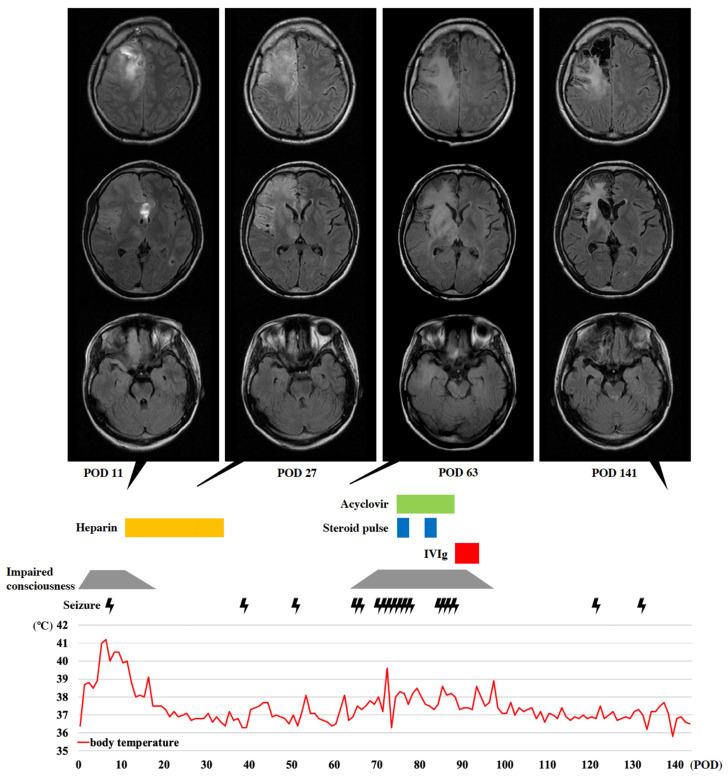
The course of clinical and MRI findings in the patient with anti-NMDAR encephalitis after CC. From POD 1, she showed fever, impaired consciousness, and left hemiplegia. On POD 11, MRI revealed a right frontal cerebral hemorrhage and right dominant bilateral frontal lobe edema with midline shift. This hyperintensity on FLAIR appeared predominantly in gray matter and also in the right orbitofrontal lobe and thalamus, but not in the pyramidal tract. Heparin therapy for CVST dramatically improved the impaired consciousness, fever, and MRI findings, such as cerebral edema and midline shift (POD 27). However, the FLAIR-hyperintense area expanded to the right precentral gyrus, basal ganglia, posterior limb of the internal capsule, and temporal lobe (POD 63). Around this time, she started to experience impaired consciousness, fever, and cluster seizure, which gradually worsened. Although steroid pulse and intravenous acyclovir did not improve these symptoms, IVIg therapy immediately improved them and reduced the FLAIR-hyperintense area (POD 141).

On postoperative day (POD) 1, CT revealed a cerebral hemorrhage on the medial side of the right frontal lobe (Figure 1B), and the patient presented with left hemiplegia. Impaired consciousness and fever were observed and gradually worsened (POD 7: Glasgow Coma Scale score [GCS], 13; body temperature, 41 °C; modified Rankin Scale [mRS], 5 [20]). CSF findings on POD 10 excluded postoperative bacterial meningitis (Table 1). Magnetic resonance angiography (MRA) and MRI revealed the disappearance of the superior sagittal sinus (Figure 1C) and right-dominant bilateral frontal lobe edema on POD 11 (Figure 2). Heparin was administered under a diagnosis of CVST. After treatment, impaired consciousness and fever promptly resolved (POD 18: GCS, 15; body temperature, <37.5 °C), and an MRI showed improvement of the cerebral edema on POD 27 (Figure 2), although left hemiplegia remained (mRS, 4). After recovery from CVST, anticoagulant therapy was continued by switching from heparin to oral warfarin.

### 2.2. Progressive Neurological Deficits (Suspected Autoimmune Encephalitis)

New symptoms such as insomnia, hallucinations, delusions, and dysphagia became slowly apparent from a month after CC. Impaired consciousness (GCS, 11) and fever of approximately 38 °C relapsed, and a new type of seizure and tachycardia (120 beats/min) were observed from 2 months postoperatively (mRS, 5). The seizure type differed from the preoperative seizure types, appearing as daily clusters of 10 or more consecutive focal impaired awareness seizures (FIASs) with conjugate deviation of the eyes to the left followed by elevation of the left arm.

The electroencephalogram showed diffuse polymorphic slowing with lower amplitude in the right frontal region and a reduction of generalized slow spike-and-slow-wave complexes found before CC, although extreme delta brush [21] was not found. The area of hyperintensity on fluid-attenuated inversion recovery (FLAIR) images gradually expanded to the right precentral gyrus, basal ganglia, thalamus, and temporopolar cortex by POD 63 (Figure 2) despite recovery from CVST.

### 2.3. Progressive Neurological Deficits Resolved after Immunotherapy

This case met the criteria for possible autoimmune encephalitis (subacute onset of altered mental status and seizures not explained by a previously known seizure) as proposed by Graus et al. [13], although CSF findings on POD 61 and 74 were mild (Table 1). Therefore, we asked another institution (Tokyo Metropolitan Institute of Medical Science) to investigate neuron surface antibodies from the serum and CSF on POD 74. Treatment with intravenous acyclovir (10 mg/kg 3 times/day for 14 days) and intravenous methylprednisolone pulse (1000 mg/day for 3 days, twice) was initiated on POD 74, on the suspicion of the possibility of both HSV encephalitis and autoimmune encephalitis. However, cluster seizures and impaired consciousness continued after these treatments. Therefore, intravenous Ig (IVIg) therapy (400 mg/kg/day for 5 days) was started on POD 89 and immediately improved the seizures and level of consciousness (GCS, 15). After completing IVIg therapy (POD 103), results for antibodies against NMDAR collected on POD 74 were revealed to be positive in both CSF and serum by cell-based indirect immunofluorescence assay (Autoimmune Encephalitis Mosaic 1; Euroimmune, Lübeck, Germany). The patient was therefore diagnosed with anti-NMDAR encephalitis after CC. CT and abdominal echography did not detect any tumors, including ovarian teratoma.

### 2.4. Outcome for This Patient

Although slight disinhibition and impulsivity became apparent as the level of consciousness improved, second-line immunosuppressive therapy with rituximab or cyclophosphamide was not administered because seizure frequency decreased to once or twice a month, and MRI showed a reduction in the area of FLAIR hyperintensity on POD 141 (Figure 2). Due to residual left hemiplegia, the patient was transferred to a rehabilitation hospital on POD 143 (GCS, 15; mRS, 4).

## 3. Discussion

This report describes a case of suspected postoperative anti-NMDAR encephalitis that sub-acutely developed various symptoms such as psychiatric symptoms, impaired consciousness, and cluster seizures and recovered from these symptoms immediately after starting immunotherapy. We suspected autoimmune encephalitis because these symptoms occurred after CVST resolved, and it met the criteria for possible autoimmune encephalitis. However, this diagnosis was challenging to make and was delayed because only one previous case report has described anti-NMDAR encephalitis after epilepsy surgery, pre-existing intellectual disability concealed psychiatric symptoms, and the CSF and MRI in this case displayed few findings showing autoimmune encephalitis. We emphasize the possibility of autoimmune encephalitis as a complication of neurosurgery even with normal CSF and MRI findings.

Clinical symptoms were the key clues to considering autoimmune encephalitis in the present case. Patients with anti-NMDAR encephalitis show a variety of acute or subacute symptoms such as psychiatric symptoms, seizures, movement disorders, impaired consciousness, central hypoventilation, and dysautonomia [12]. The predominant seizure type is focal seizure (54.5–74.2%), and status epilepticus is often observed (43.5–50%) [22,23]. In this case, seizure transformation (from spasms and tonic seizures to cluster FIASs), impaired consciousness, psychiatric symptoms (hallucination, delusion, disinhibition, and impulsivity), insomnia, and dysautonomia (tachycardia) were all considered to correspond with anti-NMDAR encephalitis [12,24]. However, psychiatric symptoms may have initially been obscured by the pre-existing intellectual disability, as in previous reports [25,26].

CSF pleocytosis in autoimmune encephalitis is considered milder than that in virus encephalitis [27]. A recent systematic review revealed that the rate of normal leukocytes on CSF testing was approximately 40% in anti-NMDAR encephalitis [28]. Thus, the presence of normal CSF leukocytes as in this case did not rule out autoimmune encephalitis. A high IgG index and positive oligoclonal band are related to the increased level of antibodies in CSF [13,29,30] and may have been suggestive of autoimmune encephalitis in the present case.

The rate of abnormal findings on an MRI is reportedly 35–50% in anti-NMDAR encephalitis, and no or only mildly abnormal MRI findings despite symptom intensity seemed to be a feature of this encephalitis [31,32,33]. This case was also judged as showing no findings other than cerebral hemorrhage and CVST at an early stage. Furthermore, the high-intensity areas on the FLAIR images for anti-NMDAR encephalitis were uncertain and variable, and abnormal findings outside the limbic system (e.g., medial temporal regions, cingulate gyrus, and insular cortex) were reportedly found in one-third of cases of anti-NMDAR encephalitis [33]. Since NMDARs are highly expressed on the neuronal surface of excitatory neurons, the predominant susceptibility of the cerebral cortex as well as the possible involvement of the basal ganglia and thalamus are considered indicative of the lesion sites in anti-NMDAR encephalitis [33,34]. Gray matter-dominant FLAIR hyperintensity at the right frontal lobe in the acute phase and at the basal ganglia and thalamus in the subacute phase (Figure 2) were also found in the present case and may be considered consistent with anti-NMDAR encephalitis.

The main triggers of anti-NMDAR encephalitis are ovarian teratomas and HSV encephalitis [12]. Apoptosis of the tumor that contains nervous tissue in the former and neuronal degeneration by HSV in the latter have been suggested to induce antigen presentation and cause anti-NMDAR encephalitis, since NMDARs are located on the neuronal cell surface [34]. Given that both triggers represent injuries to neuronal cell bodies, the same theory may be applicable to the mechanisms underlying post-craniotomy anti-NMDAR encephalitis. Namely, exposure of the antigen existing on the neuronal cell surface by injuries to the cerebral cortex during operations is considered to cause autoimmune encephalitis [19]. We speculate that the frequency of this disease might be higher than currently recognized if this theory holds true. Investigating antibodies is considered important when autoimmune encephalitis is suspected, even after neurosurgery. A system for the easy testing of neural surface antibodies needs to be established, and further cases should be accumulated.

Unfortunately, we cannot prove that this patient developed anti-NMDAR encephalitis after epilepsy surgery because of the possibility of false-positive results and previous anti-NMDAR encephalitis. However, the likelihood of false-positive results in the present case seems very low, because both the serum and CSF showed positive results for anti-NMDAR antibody. Although false-positive or false-negative results for antibodies against NMDAR can occur if only serum is used, the false-positive rate has been reported as only 0.8% when CSF is investigated [35]. Recently, the concept of autoimmune epilepsy has been advocated, and some cases of intractable epilepsy have been shown to be caused by antibodies on the neuron surface. Reports have documented that 3.6% of patients with epilepsies of unknown etiology and 4% with West syndrome showed positive results for anti-NMDAR antibodies [36,37]. The confirmation of negative results for anti-NMDAR antibodies prior to CC is thus required to demonstrate new-onset anti-NMDAR encephalitis after CC, but such evidence was not available in this case. However, we are convinced that the present patient suffered from de novo or re-activated anti-NMDAR encephalitis after surgery for the following three reasons: (1) this case met the criteria for both possible autoimmune encephalitis and anti-NMDAR encephalitis as proposed by Graus et al. [13]; (2) the results for the anti-NMDAR antibody were positive in both the CSF and serum; and (3) psychiatric symptoms, impaired consciousness, and cluster seizures improved dramatically after starting immunotherapy.

## 4. Conclusions

We documented a rare case of suspected anti-NMDAR encephalitis after CC for LGS. We propose the importance of considering autoimmune encephalitis in cases showing unexplained progressive neurological deficits after neurosurgery, even if CSF and MRI findings are mild.

## Figures and Tables

**Table 1:** Results of CSF examinations.

	POD 10	POD 61	POD 74
Pressure	NA *	17 cmH_2_O	31 cm H_2_O
Leukocytes	4/μL	3/μl	1/μL
Protein	65 mg/dl	115 mg/dl	99 mg/dl
Glucose	47 mg/dl	58 mg/dl	56 mg/dl
IgG index (reference range, <0.7)	NA *	1.239	1.03
Other findings	NA *	Oligoclonal band was positive	The antibody against NMDAR was positive. HSV-PCR and other antibodies (LGI1, Caspr2, AMPAR1, AMPAR2, and GABABR) were negative.

The results of antibodies collected on POD 74 were revealed on POD 103. * NA, not available.

## Data Availability

The data are not publicly available to ensure patient privacy. The datasets used during the current study are available from the corresponding author upon reasonable request.
